# Psychometric evaluation of the abbreviated Hungarian Faking Orgasm Scale for Women

**DOI:** 10.3389/fpsyg.2024.1513959

**Published:** 2024-12-12

**Authors:** Edit Csányi, Julia Basler, Tamás Bereczkei, Norbert Meskó

**Affiliations:** Department of Cognitive and Evolutionary Psychology, Faculty of Humanities and Social Sciences, Institute of Psychology, University of Pécs, Pécs, Hungary

**Keywords:** female faking orgasm, sexual working models, sexual motivation, scale abbreviation, relationship sexual dynamics

## Abstract

**Introduction:**

The Faking Orgasm Scale for Women (FOS) was developed to explore the motivations behind women’s self-reported instances of faking orgasm during oral sex and sexual intercourse. In a recent study, a Hungarian version of the FOS was developed, confirming the same factor structure as the original American version, consisting of four factors across two subscales.

**Methods:**

The current study aimed to develop and validate a brief Hungarian FOS. Factor analysis was conducted with data from 2220 women (mean age = 24.4, SD = 7.48 years). The Item Response Theory (IRT) analysis indicated that retaining four-four scales, each comprising of three items was the optimal solution for the revised shorter version. Validation involved 768 women (mean age = 22.6, SD = 4.54 years) completing a questionnaire package, including the Hungarian Short Form of Reasons for Having Sex Questionnaire (YSEX?-HSF) and Women’s Sexual Working Models Scale (WSWMS).

**Results:**

The results suggest that the Hungarian 24-item FOS, with its four scales within each of the two sub-scales, provides a reliable and valid measurement of motives for faking orgasm in women. The different reasons behind faking orgasm are associated with different sexual working patterns and sexual motivations. Furthermore, women who reported faking orgasms reported significantly higher levels of sexual distancing and perceived lower care from their partners compared to women who reported not faking orgasms. Women who reported faking orgasm were also more likely to engage in sexual activities to attain personal goals and cope with emotional stress.

**Discussion:**

The FOS-24 offers both practitioners and researchers a concise and useful instrument for the assessment of faking orgasms.

## 1 Introduction

The peak of sexual arousal, known as orgasm (Masters and Johnson, 1966), is marked by a range of behavioral responses and physiological processes triggered by the release of hormones (Meston et al., 2004). These processes include increased heart rate, respiratory rate, and blood pressure, as well as involuntary muscle contractions in the vagina, uterus, and fallopian tubes (Komisaruk et al., 2006). Additionally, there is a decrease in cortical activity and an increase in activity in the dopamine system, resulting in decreased cognitive functioning and intense pleasure (Georgiadis et al., 2009). Evidence suggests that the female orgasm, while not critical for fertilization (Meston et al., 2004; Wallen and Lloyd, 2008), may occur more frequently around ovulation, potentially increasing the likelihood of conception (Puts et al., 2012). This pattern has been interpreted as consistent with an evolutionary adaptivity hypothesis, although alternative explanations remain plausible. Furthermore, the experience of orgasm encompasses both psychosomatic and psychosexual processes (Mah and Binik, 2002). Weitkamp and Wehrli (2023) compared clitoral and vaginal orgasms, with clitoral orgasms described as more controllable and vaginal orgasms as deeper and more pulsating. This suggests that women’s orgasm experiences are diverse, including whole-body, cervical, and mental orgasms.

The orgasm gap refers to the disparity in orgasm frequency between men and women in heterosexual relationships, with women reporting fewer orgasms than their male partners (Frederick et al., 2017). This gap is influenced by several factors, including inadequate sexual education, limited orgasm literacy, and adherence to traditional sexual scripts that prioritize male pleasure. Sexual script theory suggests that cultural norms often shape expectations about sexual behavior, leading to unequal attention to female sexual pleasure (Gagnon and Simon, 1973). One of the major contributors to the orgasm gap is the lack of comprehensive sexual education, which often fails to address female pleasure and anatomy, leaving women less informed about their own bodies and sexual responses (Fine and McClelland, 2006). Similarly, “pleasure literacy”—the ability to understand and communicate one’s sexual needs—can be underdeveloped, contributing to the gap (Fahs and Frank, 2014).

Research indicates that men, in addition to seeking their own satisfaction, often express a desire to please their partner (Mark et al., 2014; McKibbin et al., 2010). In the absence of female partner’s orgasm (anorgasmia), men tend to evaluate themselves negatively (Salisbury and Fisher, 2014). Consequently, the impact of female orgasm extends beyond individual satisfaction to influence relationship dynamics. Female sexual satisfaction can contribute to maintaining and enhancing emotional bonds and intimacy between partners, increasing overall satisfaction, and subsequently elevating relationship satisfaction, potentially reducing the likelihood of long-term separation (Welling, 2014).

The variability of orgasmic experiences varies widely (Mah and Binik, 2001; Mangas et al., 2024a) due to individual differences in sensation, subjective evaluation, and the significance attached to one’s own orgasm (Hoy et al., 2021; Mangas et al., 2024b), playing a significant role in the lives of some individuals while holding less significance for others (Bancroft, 2008). Furthermore, gender differences are evident: unlike men, for whom satisfaction and physical pleasure are often paramount, women may not necessarily prioritize orgasm during sexual intercourse, viewing it instead as a relational factor (Cormier and O’Sullivan, 2018; Fahs and Plante, 2016; Lentz and Zaikman, 2021; Meskó et al., 2022; Salisbury and Fisher, 2014).

### 1.1 Faking orgasm

Despite the personal and interpersonal significance, they hold, orgasms are attained less consistently by women compared to men (Wallen, 2006), possibly due to women encountering greater challenges in reaching orgasm (Barnett et al., 2019). During sexual intercourse without direct clitoral stimulation, only about one-third of all women experience an orgasm (Dawood et al., 2005; Prause et al., 2016). Approximately 70% of women report using clitoral stimulation during penetration to enhance sexual pleasure (Hensel et al., 2021). Additionally, only around 32% can reach orgasm while receiving oral sex (Vannier and O’Sullivan, 2012), and 14% of women have never had an orgasm or are unsure if they have (Dunn et al., 1999; Prause et al., 2016).

Despite societal pressure that links orgasm with femininity and normalcy (Ejder Apay et al., 2013; Nicolson and Burr, 2003), which could arise from both internal and various external influences (Chadwick et al., 2019), including magazines, novels, and pornography (Cabrera and Ménard, 2013; Lavie-Ajayi and Joffe, 2009; Séguin et al., 2018), a substantial proportion of women (50–70%) choose to fake orgasms (Csányi et al., 2022; Csányi et al., 2024; Muehlenhard and Shippee, 2010). Faking the climax of sexual activity can be defined as “acting or pretending as if you have had orgasm when you have not, through vocal confirmation and/or muscular contraction, regardless of the reason” (Cooper et al., 2014, p. 426). Female participants in study Bryan’s (2001) reported that they fake their orgasms most often during penetration without clitoral stimulation and least often when the intercourse is supplemented by clitoral stimulation.

As orgasm faking is a relational phenomenon, since faking has no meaning without the presence of another person, it is associated with various relational processes such as relationship and sexual satisfaction, love, commitment, and mate retention (Bode et al., 2024; Józefacka et al., 2023; Mostova et al., 2022). While 90% of men in relationships care if their female partner experiences orgasm (McKibbin et al., 2010), nearly 70% of participating women in study Muehlenhard and Shippee’s (2010) reported faking orgasm to avoid hurting their partner’s feelings, masculinity, and ego, or to please them.

Frith (2015) suggests that faking orgasm can interpreted as a rational response to gender disparities inherent in heteronormative sexual culture. Within this framework, women are less entitled to sexual pleasure compared to men (Klein and Conley, 2022); their orgasm primarily serves to bolster their partner’s masculinity and demonstrate their commitment to them (Lentz and Zaikman, 2021). In Western cultures, particularly through media representations, there is a strong emphasis on being a “good lover” (Dormandy, 2022). For men, this entails satisfying their female partner’s needs, while for women, it involves being sexually available and enhancing the masculinity of their male partner by achieving orgasm (Potts, 2000). Consequently, women may resort to faking orgasm to prevent their partners from feeling inadequate.

In contrast, feminist perspectives argue that the unrealistic expectation for women to climax during sexual intercourse (Ejder Apay et al., 2013; Nicolson and Burr, 2003) may lead women to fake orgasm as a means of avoiding stigmatization and being perceived as abnormal (Cooper et al., 2014). Regarding oral sex, the frequency of orgasm is associated with less faking, suggesting that women are more expected to reach orgasm during intercourse than when receiving oral sex (Bryan, 2001).

### 1.2 Sexual motivation

Sexual motivation can be defined as an individual’s intention for engaging in sexual intercourse (Meston and Buss, 2007), and this desire is one of the most fundamental motivational states for both men and women (Regan and Atkins, 2006). Meston and Buss (2007) observed that sexual motivations are more diverse and psychologically complex than previously thought. In contrary to earlier research that identified only a few motivations, they discovered 237 reasons, all capable of influencing participation in sexual intercourse. These reasons span a wide spectrum; for instance, sex might serve as a resource, incentive, reward, punishment, mate-guarding strategy, or as a means to intensify the relationship (Buss, 2003; Buss and Shackelford, 1997; Meskó et al., 2022; Meston and Buss, 2007).

While sexual satisfaction tends to decrease with the length of the relationship (McNulty et al., 2016), sexual motivations also decrease markedly among women (Klusmann, 2002). This decline may be associated with menopause, during which hormonal changes can directly cause sexual dysfunctions and decrease sexual desire. Additionally, hormonal changes may exert indirect effects through psychological mechanisms such as diminished self-esteem or negative body image due to weight gain and depression (Genazzani et al., 2007).

Research on sexual motivations has also revealed gender differences in attitudes toward sex and sexual behaviors (Meston and Buss, 2007). Generally, women’s sexual motivation tends to be more emotional, while men are more motivated by purely physical reasons to engage in sexual activities that may not require a deep understanding of the partner (Basson, 2000). Men tend to masturbate, consume pornographic content, and have sexual thoughts more frequently than women (Petersen and Hyde, 2010). Meskó et al. (2022) highlighted gender-specific patterns in sexual motivations, noting that men are driven by personal goals, leading them to be more impulsive and seek novelty, potentially resulting in infidelity more often.

In contrast, women’s sexual motivation is more characterized by coping with emotional difficulties, such as fear of abandonment, which may be interpreted as a form of subordination (Barbaro et al., 2015). Women are more likely to engage in sexual intercourse because they believe it will help maintain their partner, increase commitment to the relationship, and provide emotional closeness, bonding, and feelings of love (Impett et al., 2005).

Lehmiller (2023) suggests that sexual attitudes and behaviors are influenced not only by biological determinism but also by psychosocial factors, including societal expectations to conform to norms. Additionally, social pressures can also manifest in the perception of sexual availability as a commodity that can be exchanged for goods or resources (Meskó et al., 2022).

The Hungarian Short Form of Reasons for Having Sex Questionnaire (YSEX?-HSF; Meskó et al., 2022) was chosen for validating the Faking Orgasm Scale because it effectively captures a wide range of sexual motivations, including those focused on coping strategies. This focus on coping-driven sexual motivation is particularly relevant, as it likely relates to the reasons women may fake orgasms, providing deeper insight into the psychological factors influencing this behavior.

### 1.3 Women’s sexual working models

Although sexuality is typically understood within a relational framework, it is essential to consider not only general characteristics but also individual differences that influence the psychological functioning of the sexual system including associated motivations, emotions, and cognition when examining sexual activities (Birnbaum et al., 2014; Meskó and Õry, 2023). Birnbaum et al. (2014) developed a multidimensional assessment to evaluate experiences of heterosexual intercourse in women, providing a multifaceted emotional, cognitive, and motivational profile of women’s sexuality. These models of sexual functioning reveal individual patterns of attitudes, responses and behaviors within women’s sexual activities, helping to define the role of sexuality in women’s lives and enhancing comprehension of the complexities of female sexual behavior (Birnbaum et al., 2001). Additionally, they may unveil dyadic relationship dynamics, such as the connection between sexual activity and relationship satisfaction (Hassebrauck and Aron, 2001).

However, sexual functioning can also involve contradictory elements, as highlighted by Birnbaum and Reis (2006), where a woman may experience negative emotions during sexual activities despite believing in the importance of sex in maintaining her relationship. Women with such ambivalent sexual experiences may behave differently from those who have not encountered such experiences (Birnbaum, 2003). Therefore, to fully comprehend the intricate functioning of sexuality, models of sexual functioning must encompass various positive and negative emotions, affective responses to the sexual cycle, as well as thoughts and goals related to the self, the partner, the dyadic relationship, and the sexual act (Birnbaum et al., 2016).

The Women’s Sexual Working Models Scale (WSWMS; Birnbaum et al., 2014) was chosen for validating the Faking Orgasm Scale for Women (FOS) because it provides a comprehensive assessment of women’s internalized beliefs and expectations about sexual relationships. Its relevance lies in its ability to capture the psychological and relational dynamics that influence sexual behavior, making it a valuable tool for understanding the underlying motivations and attitudes that may drive behaviors like faking orgasms.

Over the last decade, there has been relatively limited research employing the original Faking Orgasm Scale for Women (Cooper et al., 2014), probably due to its extensive length (56 items). Compared to longer scales, concise questionnaires may be preferable for several reasons. Although longer scales can offer more extensive data, they also tend to induce respondent fatigue, increase response error rates, and reduce completion rates (Rolstad et al., 2011; Saucier, 1994). Furthermore, it is believed that shortening scales may have less impact on psychometric quality due to the reduction of redundancy. As a result, more condensed self-report instruments may exhibit even stronger validity indices (Burisch, 1997). Therefore, the development of short and multidimensional questionnaires with high psychometric properties benefits both researchers and participants (Jonason and Webster, 2010).

## 2 Research aim

The aim of the present study was twofold. First, we sought to reduce the number of items in the Hungarian version of the Faking Orgasm Scale for Women (FOS; Csányi et al., 2024) to develop a shorter instrument while maintaining the ability to assess the four major dimensions of orgasm faking during oral sex and sexual intercourse, as captured by the original version. The secondary aim was to analyze the criterion validity of the FOS-24 to ensure the psychometric quality of the shorter scale and create a more concise measure of orgasm faking among women for studies requiring data on the fundamental patterns of faking orgasm. Additionally, we evaluated whether there is a correlation in motivation behind orgasm faking with questionnaires on sexual working models and motivation for participating in sexual activities, which are key factors in sexuality. We anticipated that the newly developed instrument would exhibit similar correlations with sexual motivations (YSEX?-HSF; Meskó et al., 2022) and the Women’s Sexual Working Models Scale (WSWMS; Birnbaum et al., 2014) as observed by Csányi et al. (2024).

## 3 Materials and methods

### 3.1 Participants and procedure

Data from Csányi et al. (2024) were utilized for the abbreviation and validation procedure of the FOS-24.

**Sample 1**

The factor analysis was conducted using responses from a sample of 2,220 individuals, all identifying their birth sex as female and current gender identity as woman. The average age of the sample was 24.40 years (SD = 7.48, min = 18 years, max = 80 years). Of these participants, 1,726 (77.7%) reported being in some form of relationship at the time of the study; 849 were in a relationship, 677 were in a cohabitation relationship with a partner, and 200 stated that they were married. Among the respondents, 752 (33.9%) reported faking orgasm during receiving oral sex, while 1,051 (47.3%) reported faking orgasm during sexual intercourse at least once in their life. Specifically, 155 participants reported faking orgasm only during receiving oral sex, 454 reported faking orgasm only during sexual intercourse, and 597 reported faking it in both situations.

**Sample 2**

A total of 768 women completed the questionnaire package used for validation. In addition to demographic questions, they completed three questionnaires investigating faking orgasm in two scenarios, psychological motives associated with women’s sexual working models, and motivations behind engaging in sexual intercourse. The age of participants ranged from 18 to 48 years (*M* = 22.6 years, SD = 4.54). Of these participants, 552 (71.9%) were in some form of relationship (dating, cohabitation, marriage). Among respondents, 278 (36.2%) reported faking orgasm while receiving oral sex, and 369 (52%) reported faking orgasm during sexual intercourse at least once in their life. 53 participants reported faking orgasm during oral sex only, 144 during sexual intercourse only, and 225 reported faking it in both situations. Information on sexual orientation was not collected in this survey. Therefore, it can be assumed that, as in previous Western surveys (e.g., Bailey et al., 2016; Ganna et al., 2019), predominantly same-sex attracted respondents make up less than 5% of the respondents in this sample.

All respondents filled out the questionnaires online, using Qualtrics. The link to the survey was disseminated via social media sites (e.g., Facebook, Instagram) and university mailing lists. Our goal was to obtain a heterogeneous sample and so we intentionally ensured that the survey was accessible to various segments of the population. All participants gave informed consent, and none of them were rewarded for participation. The studies received ethical approval from the Hungarian United Ethical Review Committee for Research in Psychology (Ref. No. 2017/21, 2022/107). The studies were not preregistered. *A priori* sample size calculation was not performed. All source data are available at: https://osf.io/96emq/?view_only=8eca7faedc0d4f67848c029eac2a0070.

### 3.2 Measures

As one questionnaire was not yet available in Hungarian the authors initially translated the items and instructions of the Women’s Sexual Working Models (WSWMS; Birnbaum et al., 2014) into Hungarian. The resulting Hungarian version underwent verification using the standard back-translation technique (Brislin, 1980). This involved retranslating the items and instructions back into English by an independent translator not associated with the study. Any discrepancies that arose during the back-translation process were resolved by the two translators. Following the validation process established by Csányi et al. (2024) for the 56-item version of the Hungarian FOS, we utilized the same measures, including the Short Form of Reasons for Having Sex Questionnaire (YSEX-HSF; Meskó et al., 2022) and Women’s Sexual Working Models (WSWMS; Birnbaum et al., 2014). This approach aimed to improve comparability between the newly developed shorter version and the longer version of FOS.

#### 3.2.1 Faking Orgasm Scale for Women, Hungarian form (FOS)

The FOS [ Cooper et al., 2014; Hungarian version developed by Csányi et al. (2024)] is a comprehensive 56-item self-report tool designed to explore women’s motivations for faking orgasm during sexual intercourse (33 items; sexual intercourse subscale) and while receiving oral sex (23 items; oral sex subscale). Participants provided responses to open-ended questions and indicated their level of agreement with each item on a 5-point scale ranging from Never to Always. Only participants who reported faking orgasm during sexual intercourse completed the Sexual Intercourse subscale. This subscale encompasses four dimensions: Altruistic Deceit (SIAD), which assesses the respondent’s concern for their partner’s feelings as a motivation for faking orgasm (e.g., “To make your partner happy”); Fear and Insecurity (SIFI), which measures whether women fake orgasm to avoid negative emotions (e.g., “Because you are ashamed you cannot reach orgasm”); Elevated Arousal (SIEA), which evaluates women’s tendency to use faked orgasm to heighten their arousal during sexual intercourse (e.g., “To turn yourself on”); and Sexual Adjournment (SISA), referring to faking orgasm as a means to quickly end sexual intercourse (e.g., “Because you simply are not enjoying yourself”). Likewise, only participants who reported faking orgasm during oral sex completed the Oral Sex subscale. This subscale also had four dimensions: Altruistic Deceit (OSAD), Insecure Avoidance (OSIA), Elevated Arousal (OSEA), and Fear of Dysfunction (OSFD). These dimensions assess various motivations for faking orgasm during oral sex, including concern for the partner’s feelings, avoidance of negative emotions, arousal enhancement, and fear related to sexual health or inadequate response. Internal reliability indicators of the questionnaire are presented in Table 1.

**TABLE 1:** The internal reliability indicators of the questionnaires.

Measures	Subscales	McDonald’ ω
**Faking Orgasm Scale for Women, Hungarian brief form (FOS-24)**		
Oral sex		0.788
	Altruistic deceit	0.901
	Insecure avoidance	0.808
	Elevated arousal	0.821
	Fear of dysfunction	0.800
Sexual intercourse		0.757
	Altruistic deceit	0.851
	Fear and insecurity	0.845
	Elevated arousal	0.909
	Sexual adjournment	0.749
**Women’s Sexual Working Models Scale (WSWMS)**		
	Guilt and shame	0.903
	Maintain the bond	0.883
	Distancing/distraction	0.889
	Caring partner	0.894
	Excitement	0.826
**Reasons for Having Sex Questionnaire Hungarian Short Form (YSEX?-HSF)**		
	Personal goal attainment	0.897
	Relational reasons	0.923
	Sex as coping	0.902

#### 3.2.2 Women’s Sexual Working Models Scale (WSWMS)

The WSWMS developed by Birnbaum and Reis (2006), is a self-report instrument designed to assess various dimensions of feelings, expectations, and beliefs regarding sexual activity. It provides a reliable measure of cognitive, behavioral, and affective aspects of individuals’ sexual lives within romantic relationships. This scale outlines five key dimensions of sexual behavior that contribute uniquely to understanding sexuality in women’s romantic relationships. (1) The Guilt and Shame factor relates to negative sexual self-perception and anxiety, and it is inversely correlated with sexual satisfaction (e.g., “Engaging in sexual activity makes me feel guilty”). (2) Maintain the Bond reflects the belief that sexual interactions foster intimacy between partners and strengthens their emotional connection (e.g., “Sexual activity serves to deepen the bond between two individuals”). (3) Distancing/Distraction signifies feelings of detachment from the sexual experience and one’s partner due to intrusive thoughts (e.g., “During sexual activity my mind is often preoccupied with distracting thoughts”). (4) Caring Partner assesses the perception of a sexual partner as attentive and responsive to one’s needs during sexual encounters (e.g., “My partner demonstrates care and consideration for me during sexual activity”). (5) Excitement represents intense sexual desire, a powerful motivator of human behavior (e.g., “During sexual activity, I experience a strong sense of excitement”). Participants rated each statement on a 5-point scale (1 = Somewhat characteristic, 5 = Very characteristic) based on its relevance to their experiences. Internal reliability indicators for the questionnaire are detailed in Table 1.

#### 3.2.3 Reasons for having sex questionnaire, Hungarian form (YSEX?-HSF)

The YSEX?-HSF (Meskó et al., 2022) is a self-report instrument which includes three subscales for measuring sexual motivation. Based on the original American YSEX? questionnaire (Meston and Buss, 2007), Meskó et al. (2022) created the Hungarian version of the YSEX? questionnaire, which included three factors instead of the original four-factor structure. The YSEX?-HSF is comprised of three subscales with 73 items. The Personal Goal Attainment subscale includes reasons that lead an individual to engage in sexual intercourse to achieve personal sexual interests (e.g., “I wanted to seek experience”). The Relational Reasons subscale refers to reasons that lead an individual to have sexual intercourse because some aspect of the partner relationship is important (e.g., “I wanted to celebrate”). The Sex as Coping subscale refers to reasons that lead an individual to have sexual intercourse as a way of coping with internal (personal) or external (relational) conflicts (e.g., “I wanted to retain the relationship”). Participants were requested to indicate how frequently each of the listed reasons led them to have sexual intercourse in the past. If someone had not yet had sex, they were asked to use the scale to indicate what the likelihood that each of the listed reasons would lead them to have sex. Each item was rated on a 5-point scale: “None of my sexual experiences” (1), “A few of my sexual experiences” (2), “Some of my sexual experiences” (3), “Many of my sexual experiences” (3), “All of my sexual experiences” (5). Higher scores indicate higher sexual motivation. The internal reliability indicators of the questionnaire are presented in Table 1.

### 3.3 Statistical analyses

We used a confirmatory factor analysis (CFA) first to confirm the unidimensionality of the latent variables, as that is a requirement of item response theory (IRT). We used the diagonally weighted least squares (DWLS) estimator. To evaluate model fit, we used the comparative fit index (CFI), Tucker-Lewis index (TLI), the root mean square error of approximation (RMSEA), and the standardized root mean squared residual index (SRMR). Cutoff values indicative of good model fit were CFI and TLI values of 0.95 or greater (Hu and Bentler, 1998; Babyak and Green, 2010), and RMSEA and SRMR values of 0.06 or lower (Babyak and Green, 2010).

In our analysis of the psychometric properties of each item, we employed the graded response model (GRM) developed by Samejima (1968). The GRM is particularly suited for ordinal variables such as Likert-type scales, as it accounts for varying levels of agreement across response categories. This model allows items to relate differently to a latent trait (i.e., the different dimensions of faking orgasms). Our focus was on discrimination parameter (*a*), which shows the slope of the scale at a given item location; a steeper slope indicates a better discrimination property of the item (Baker, 2001). A higher *a* value indicates that the item is more effective at distinguishing between individuals with similar levels of the latent trait. We retained the best items among the ones with very high discrimination ability (*a* > 1.7; Baker, 2001), as these items allow to discriminate between individuals precisely even in an abbreviated scale.

Next, we calculated McDonald’s ω coefficients to check the internal consistency of each questionnaire we used. We chose the McDonald’s omega over Cronbach’s alpha because it allows a more accurate measure of internal consistency when the assumptions of the tau-equivalent model (i.e., violation of the equal-item variance) are not met (Dunn et al., 2014).

To evaluate the psychometric properties of the FOS-24, a confirmatory factor analysis (CFA) was performed for the two scales separately. The same model fit indicators as above described were used. The external validity of the FOS-24 was tested with Spearman’s coefficients to examine the correlations between each subscale of the two scales and each subscale of the WSWMS, YSEX?-HSF and the original version of the Hungarian FOS. Due to violations of normal distribution, Spearman correlations were used. The Benjamini-Hochberg false discovery rate (FDR) procedure was applied to correct for multiple testing (Benjamini and Hochberg, 1995; Verhoeven et al., 2005) with *p-*values accepted at an FDR-corrected threshold of *q* < 0.05. This method offers a more balanced approach as opposed to traditional and conservative methods (like the Bonferroni correction) and controls the proportion of false positives among significant results. The Benjamini-Hochberg corrected *p*-values were calculated using the spreadsheet available at http://www.biostathandbook.com/benjaminihochberg.xls. In our manuscript we report the p-values that remained significant after correction.

Differences between faking and non-faking women were analyzed using Mann-Whitney U tests, as the variables were not normally distributed.

## 4 Results

### 4.1 Item response analysis

Confirmatory factor analyses were run to confirm unidimensionality. For both scales, the results indicated that the four-four factor model provided an acceptable fit (Oral subscale: CFI = 0.985, TLI = 0983, RMSEA = 0.084 [90% CI: 0.079–0.089], SRMR = 0.096; Intercourse subscale: CFI = 0.983, TLI = 0.982, RMSEA = 0.081 [90% CI: 0.079–0.084], SRMR = 0.091). As the CFAs confirmed that all scales had a single latent variable, the FOS-24 was analyzed using GRM IRT.

First, we examined the Oral subscales. For Altruistic Deceit subscale, eight out of nine items (6, 7, 8, 9, 10, 11, 12, 13) on the scale met the *a priori* threshold (1.7) for the parameter *a*. We decided to retain the three items with the highest parameter *a* value, items 7, 8, and 9, respectively. On the Insecure Avoidance subscale, three out of four items (1, 2, 4) met the threshold of 1.7, so we retained all three items. On the Elevated Arousal subscale, three out of five items (16, 17, 18) met the threshold, all of which we retained. On the Fear of Dysfunction subscale, three out of four items (15, 21, 22) met the necessary a value, so we retained them all. Thus, the shortened Oral subscales consist of 12 items, three for each subscale.

Next, the Intercourse subscales was analyzed. For Altruistic Deceit, 11 out of 14 items (1, 2, 3, 4, 5, 8, 9, 11, 12, 14, 16) met the *a priori* threshold of 1.7 for parameter *a*, and we decided to retain the three items with the highest *a* value (items 3, 4, and 9). For the Fear and Insecurity subscale, eight out of ten items (7, 21, 22, 23, 24, 25, 26, 27) reached the threshold, of which we selected the three with the highest values (7, 22, 25). On the Elevated Arousal subscale, five out of seven items (29, 30, 32, 33, 34) had a parameter a value higher than 1.7, of which the retained items (32, 33, 34) had the highest. Finally, on the Sexual Adjournment subscale, all three items (15, 17, 19) reached the necessary threshold and were retained for the short version of the questionnaire. Thus, the shortened Intercourse subscales consist of 12 items, three for each subscale.

Details of parameter *a* values for all items are provided in Supplementary Table 1. The Supplementary Materials also include the final version of the FOS-24 with a short scoring guide in Hungarian.

### 4.2 Properties of the abbreviated scales

To assess the psychometric properties of the FOS-24, we conducted confirmatory factor analysis (CFA) for each scale separately. For both scales, the results indicated that the four-four factor model provided an acceptable fit (Oral subscale: CFI = 0.991, TLI = 0.988, RMSEA = 0.029 [90% CI: 0.016–0.040], SRMR = 0.039; Intercourse subscale: CFI = 0.984, TLI = 0.978, RMSEA = 0.038 [90% CI: 0.0296–0.046], SRMR = 0.04; Factor loadings are provided in Supplementary Table 2).

Based on the McDonald’s omega, both scales of the brief version showed adequate internal consistency, similar to the other used questionnaires (WSWMS, YSEX?-HSF; for further details, refer to Table 1). The values for the shortened version were comparable to those of the original scales.

### 4.3 External validity of the abbreviated scales

The external validity was analyzed by calculating the correlations between each subscale of the two main scales of FOS-24 with the original version of the Hungarian FOS (see Table 2), as well as each subscale of the WSWMS (Table 3) and YSEX?-HSF (Table 4). The results indicate that the correlations of FOS and FOS-24 were highly similar with the sexual working models and sexual motivations.

**TABLE 2:** Spearman correlation between 24-item and 56-item versions of Hungarian Faking Orgasm Scale for Women.

FOS scales	Spearman *r*
Oral sex subscales	
Altruistic deceit (OSAD)	0.915***
Insecure avoidance (OSIA)	0.934***
Elevated arousal (OSEA)	0.977***
Fear of dysfunction (OSFD)	0.989***
Sexual intercourse subscales	
Altruistic deceit (SIAD)	0.898***
Fear and insecurity (SIFI)	0.922***
Elevated arousal (SIEA)	0.982***
Sexual adjournment (SISA)	1.000***

***p < 0.001.

**TABLE 3:** Spearman correlation between reasons for faking orgasm (FOS-24, FOS-56) and sexual working models (WSWMS).

Women’s Sexual Working Models Scale (WSWMS)	Hungarian Brief version of Faking Orgasm Scale for Women (FOS-24)				56-item version of the Hungarian Faking Orgasm Scale for Women (FOS; Csányi et al., 2024)			
	**Oral Sex subscales**							
	**OSAD**	**OSIA**	**OSEA**	**OSFD**	**OSAD**	**OSIA**	**OSEA**	**OSFD**
Guilt and shame	0.079	0.211***	0.085	0.158**	0.089	0.232***	0.117	0.170**
Maintain the bond	0.106	0.003	0.192**	0.050	0.135*	0.040	0.187**	0.039
Distancing	0.176 **	0.268 ***	0.050	0.327 ***	0.188**	0.297***	0.057	0.341***
Caring partner	−0.044	−0.060	0.042	−0.164 **	−0.037	−0.059	0.014	−0.171**
Excitement	−0.103	−0.199 ***	0.247 ***	−0.121 *	−0.051	−0.189**	0.239***	−0.132*
	**Sexual Intercourse subscales**							
	SIAD	SIFI	SIEA	SISA	SIAD	SIFI	SIEA	SISA
Guilt and shame	0.060	0.134 **	0.104 *	0.206 ***	0.081	0.212***	0.092	0.206***
Maintain the bond	0.179 ***	0.019	0.155 **	−0.087	0.179***	0.022	0.137**	−0.087
Distancing	0.150 **	0.319 ***	0.070	0.385 ***	0.208***	0.353***	0.069	0.385***
Caring partner	−0.068	−0.166 **	−0.035	−0.268 ***	−0.093	−0.198***	−0.035	−0.268***
Excitement	−0.04	−0.098	0.159 **	−0.187 ***	−0.009	−0.111*	0.175***	−0.187***

*p < 0.05; **p < 0.01; ***p < 0.001; Oral Sex subscales: Altruistic Deceit (OSAD); Insecure Avoidance (OSIA); Elevated Arousal (OSEA); Fear of Dysfunction (OSFD); Sexual Intercourse subscales: Altruistic Deceit (SIAD); Fear and Insecurity (SIFI); Elevated Arousal (SIEA); Sexual Adjournment (SISA); WSWMS, Women’s Sexual Working Models Scale.

**TABLE 4:** Spearman correlation between reasons for faking orgasm (FOS-24, FOS-56) and sexual motivations (YSEX?-HSF).

		Hungarian Brief version Faking Orgasm Scale for Women (FOS-24)				56-item version of the Hungarian Faking Orgasm Scale for Women (FOS; Csányi et al., 2024)			
		**Oral Sex subscales**							
		**OSAD**	**OSIA**	**OSEA**	**OSFD**	**OSAD**	**OSIA**	**OSEA**	**OSFD**
Reasons for Having Sex Questionnaire, Hungarian form (YSEX-HSF)	Personal Goal Attainment	0.08	0.037	0.192 **	0.123 *	0.108	0.044	0.206***	0.120*
	Seeking novelty	0.056	−0.033	0.078	0.074	0.048	−0.053	0.083	0.07
	Conformity	0.026	0.128 *	0.012	0.062	0.052	0.137*	0.006	0.067
	Infidelity	−0.01	−0.068	0.012	−0.009	−0.038	−0.074	0.021	−0.01
	Impulsiveness	−0.05	0.069	0.155 **	0.150 *	−0.027	0.073	0.187**	0.144*
	Revenge	0.004	0.016	0.034	−0.009	0.024	0.055	0.051	−0.001
	Seeking sensation	0.087	−0.091	0.098	−0.015	0.082	−0.086	0.108	−0.019
	Control and power	0.122 *	0.039	0.115	−0.017	0.108	0.038	0.131*	−0.027
	Boosting self-esteem	0.115	0.151 *	0.235 ***	0.239 ***	0.169**	0.175**	0.245***	0.245***
	Relational reasons	0.093	−0.023	0.158 **	0.043	0.123*	0.023	0.158**	0.038
	Sexual desire	0.058	−0.053	0.072	−0.004	0.061	−0.046	0.066	−0.014
	Commitment	0.093	0.008	0.095	0.038	0.129*	0.044	0.089	0.041
	Physical attraction	0.128 *	−0.041	0.049	0.013	0.103	−0.014	0.05	0.009
	Relaxation	0.045	−0.068	0.164 **	0.061	0.06	−0.021	0.194**	0.056
	Intimacy	0.053	0.039	0.123 *	−0.023	0.073	0.065	0.102	−0.02
	Excitement	0.05	−0.136 *	0.046	−0.045	0.04	−0.126*	0.063	−0.051
	Self-affirmation	0.058	0.03	0.152 *	0.039	0.101	0.056	0.151*	0.031
	Care	0.03	−0.016	0.063	0.101	0.068	0.026	0.059	0.098
	Happiness seeking	0.048	0.065	0.170 **	0.138 *	0.119*	0.1	0.153*	0.133*
	Sex as coping	0.161 **	0.114	0.228 ***	0.197 ***	0.173**	0.141*	0.236***	0.196***
	Mitigating emotional deficit	0.125 *	0.114	0.217 ***	0.150 *	0.145*	0.156**	0.237***	0.157**
	Compulsion and avoidance	0.189 **	0.123 *	0.137 *	0.188 **	0.146*	0.155**	0.125*	0.194**
	Utilitarianism	−0.009	0.003	0.129 *	0.002	0.022	−0.02	0.144*	−0.007
	Coping with relational conflicts	−0.057	0.079	0.130 *	0.107	−0.066	0.057	0.125*	0.099
	Submissiveness	0.154 *	0.175 **	0.216 ***	0.194 **	0.205***	0.194**	0.239***	0.191**
	Coping with partner’s emotional demands	0.137 *	−0.044	0.007	−0.058	0.101	−0.016	0.023	−0.061
	Mate retention	0.178 **	0.091	0.109	0.163 **	0.173**	0.111	0.117	0.162**
Reasons for Having Sex Questionnaire, Hungarian form (YSEX-HSF)	Personal Goal Attainment	0.116 *	0.07	0.186 ***	0.223 ***	0.155*	0.124*	0.185***	0.223***
	Seeking novelty	0.055	0.075	0.116 *	0.143 **	0.09	0.094	0.122*	0.143**
	Conformity	0.105 *	0.09	0.003	0.152 **	0.105*	0.103*	0.021	0.152**
	Infidelity	−0.017	−0.088	0.089	0.156 **	0.004	−0.026	0.076	0.156**
	Impulsiveness	0.02	0.054	0.145 **	0.109 *	0.036	0.063	0.144**	0.109*
	Revenge	0.019	−0.036	0.109 *	0.120 *	0.065	0.019	0.114*	0.120*
	Seeking sensation	0.083	−0.064	0.079	0.185 ***	0.061	−0.024	0.064	0.185***
	Control and power	0.029	−0.014	0.105 *	0.022	0.071	0.021	0.112*	0.022
	Boosting self-esteem	0.166 **	0.220 ***	0.258 ***	0.175 ***	0.232***	0.273***	0.258***	0.175***
	Relational Reasons	0.193 ***	0.143 **	0.259 ***	0.03	0.225***	0.162**	0.262***	0.03
	Sexual desire	0.126 *	−0.012	0.043	−0.017	0.124*	−0.019	0.038	−0.017
	Commitment	0.156 **	0.125 *	0.169 **	−0.014	0.179***	0.144**	0.165**	−0.014
	Physical attraction	0.126 *	0.06	0.135 **	0.035	0.106*	0.078	0.149**	0.035
	Relaxation	0.075	0.031	0.170 **	0.135 **	0.088	0.067	0.180***	0.135**
	Intimacy	0.1	0.058	0.07	−0.107 *	0.081	0.023	0.076	−0.107*
	Excitement	0.101	0.057	0.169 **	−0.008	0.111*	0.07	0.166**	−0.008
	Self-affirmation	0.157 **	0.169 **	0.303 ***	0	0.240***	0.175***	0.303***	0
	Care	0.185 ***	0.175 ***	0.204 ***	0.106 *	0.227***	0.201***	0.205***	0.106*
	Happiness seeking	0.188 ***	0.193 ***	0.249 ***	−0.014	0.228***	0.215***	0.242***	−0.014
	Sex As Coping	0.203 ***	0.222 ***	0.252 ***	0.351 ***	0.253***	0.302***	0.255***	0.351***
	Mitigating emotional deficit	0.134 **	0.220 ***	0.211 ***	0.261 ***	0.203***	0.278***	0.219***	0.261***
	Compulsion and avoidance	0.149 **	0.122 *	0.106 *	0.422 ***	0.155**	0.148**	0.099	0.422***
	Utilitarianism	0.012	−0.03	0.157 **	0.076	0.024	0.018	0.137**	0.076
	Coping with relational conflicts	0.05	0.081	0.141 **	0.125 *	0.079	0.131*	0.147**	0.125*
	Submissiveness	0.181 ***	0.231 ***	0.211 ***	0.250 ***	0.238***	0.298***	0.214***	0.250***
	Coping with partner’s emotional demands	0.143 **	0.067	0.059	0.272 ***	0.159**	0.074	0.074	0.272***
	Mate retention	0.203 ***	0.190 ***	0.192 ***	0.291 ***	0.225***	0.269***	0.185 ***	0.291 ***

*p < 0.05; **p < 0.01; ***p < 0.001; Oral Sex subscales: Altruistic Deceit (OSAD); Insecure Avoidance (OSIA); Elevated Arousal (OSEA); Fear of Dysfunction (OSFD); Sexual Intercourse subscales: Altruistic Deceit (SIAD); Fear and Insecurity (SIFI); Elevated Arousal (SIEA); Sexual Adjournment (SISA); YSEX?-HSF, Hungarian Short Form of Reasons for Having Sex Questionnaire.

### 4.4 Comparison between women who reported faking orgasm and women who did not

**FIGURE 1:**
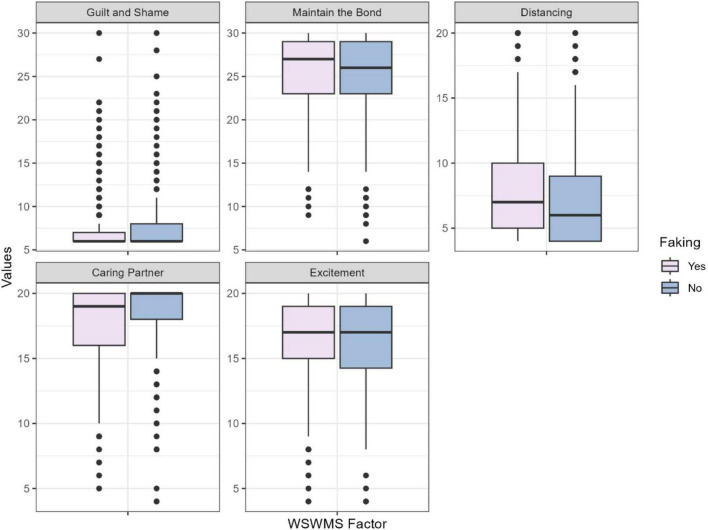
Sexual working models of women who reported having ever faked an orgasm compared to women who reported having never faked an orgasm (*N* = 768). WSWMS, Women’s Sexual Working Models Scale.

**FIGURE 2:**
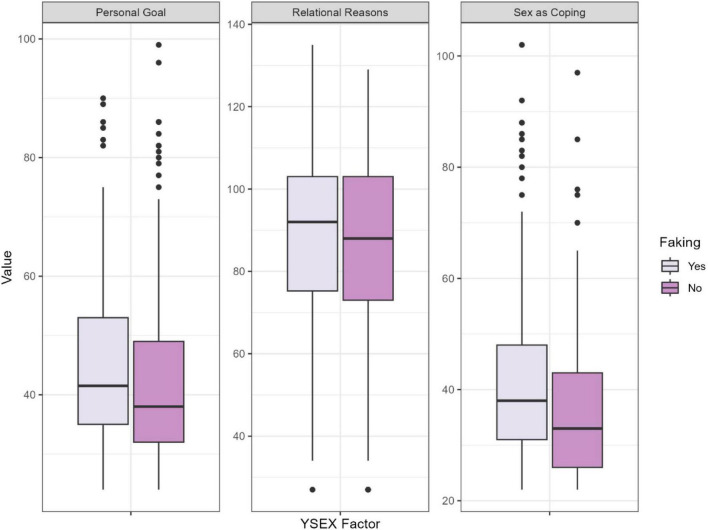
Sexual motivation of women who reported having ever faked an orgasm compared to women who reported having never faked an orgasm (*N* = 768). YSEX, Reasons for Having Sex Questionnaire.

Using Mann-Whitney tests, we investigated whether there are differences in sexual functioning models (see Figure 1) and sexual motivations (see Figure 2) between women who reported faking orgasm and those who did not, both when receiving oral sex and during sexual intercourse. Among the different sexual functioning models, there is a significant difference in the Distancing and Caring Partner dimensions both during receiving oral sex and sexual intercourse (See the results of the Mann Whitney U test and the average scores of the factors in Supplementary Table 3) The differences in the mean scores suggest that women who fake orgasm are more inclined to maintain distance in sexual situations due to experienced indifference and detachment from the sexual event and the partner. Moreover, they perceive their sexual partners as caring for their needs to a lesser extent. This factor can predict both sexual and relational satisfaction (Birnbaum and Reis, 2006), indicating that those who do not perceive their partner’s care are less likely to experience satisfaction and may resort to faking orgasm.

Regarding the primary sexual motivations in both examined situations, based on mean scores, it is more characteristic of women who fake orgasm to engage in sexual activities to achieve personal goals and use sex as coping mechanism.

## 5 Discussion

### 5.1 Development of the brief version

The primary aim of the study was to develop a shortened form of the Hungarian version of the Faking Orgasm Scale for Women (FOS) (Csányi et al., 2024). We employed Item Response Theory (IRT) to identify items with optimal discrimination parameters. Crucially, this abbreviated Hungarian version of the FOS retains the informational integrity of the original questionnaire concerning its four factors across both scales (Sexual Intercourse and Oral Sex). The final three-item short forms of the Hungarian FOS-24 subscales exhibited excellent reliability and internal consistency. Significantly, all subscales exhibited a positive shift on the latent trait, indicative of the instrument’s validity and effectiveness in examining potential motives across diverse samples. In summary, the FOS-24 demonstrates robust psychometric properties, offering valid and reliable assessments of the twice-four motivations for faking orgasm while accommodating individual differences within our Hungarian sample.

### 5.2 External validity assessment

The secondary aim of the present study was to evaluate the external validity of the FOS-24 using self-report measures of motivations and models of sexuality.

#### 5.2.1 Women’s sexual working models scale

Consistent with the findings of the 56-item version of the FOS (Csányi et al., 2024), the strongest associations with faking orgasm were found with the Distancing factor of the Women’s Sexual Working Models Scale (Birnbaum and Reis, 2006) in both sexual situations examined (during sexual intercourse and receiving oral sex). This suggests that orgasm faking, driven by negative emotions such as fear, uncertainty, and sexual postponement, leads to emotional indifference toward the partner and the sexual encounter, significantly contributing to a decreased relationship satisfaction among women who reported faking orgasm (Csányi et al., 2022). The strongest correlation is observed with orgasm faking for the purpose of Sexual Adjournment, which may serve two functions. It allows the individual to shorten the duration of unpleasant sexual activity, while also avoiding relationship tensions that might result from directly rejecting the partner’s sexual advances (Thomas et al., 2017). Thus, overall, it can be viewed as a defense mechanism (Frith, 2018), contributing to increased relational and sexual detachment.

Perceiving the partner as caring and sensitive to one’s needs showed a significant, moderately strong negative correlation only with orgasm faking for the purpose of delaying sexual intercourse. This finding is consistent with Frith’s (2018) explanation, which suggests that due to a partner’s emotional distance, lack of sympathy, and limited potential for honest communication, faking orgasm becomes one of the least risky ways to terminate sexual activity.

During receiving oral sex, behaviors associated with negative self-schema and sexual anxiety showed positive associations with faking orgasm as a means of avoiding one’s own negative emotions. These behaviors often stem from feelings of shame and guilt arising from conflicts with body image and genital self-image (Hoy et al., 2021). The exposure of the vulva (external genitalia) during oral sex may exacerbate anxiety and shame related to dissatisfaction with them (Fahs, 2014), thus prompting individuals to fake orgasm.

Media influence may contribute to the association between faking and feelings of shame during sexual intercourse, as it frequently portrays reaching orgasm as the primary indicator of sexual satisfaction (Lavie-Ajayi and Joffe, 2009). Sociocultural norms also exert pressure, suggesting that experiencing orgasm is essential for feeling normal and feminine (Nicolson and Burr, 2003), thereby imposing both internal and external pressure to achieve orgasm (Chadwick et al., 2019). The absence of orgasm can lead to stigma and an increased experience of unpleasant, negatively valenced emotions (Fahs and Swank, 2016; Lavie-Ajayi and Joffe, 2009). Therefore, many individuals resort to faking orgasm to alleviate shame and swiftly conclude sexual activity.

By adopting a self-perception perspective during orgasm faking, women may observe their behavior as an external observer, potentially enhancing sexual arousal (Barnett et al., 2019). This pattern was evident in our sample, with a significant association found between the Excitement factor, indicative of intense sexual desire, and faking orgasm to elevate arousal levels, particularly during receiving oral sex.

In the case of women who reported that sexual activity enhances the relationship between partners, our analysis revealed only negligible, albeit statistically significant, correlations with various motivations for faking orgasm (i.e., *r* < | 0.2|; Ferguson, 2009). Although Séguin et al. (2018) suggests that faking orgasm for romantic reasons may benefit the relationship, our study does not support this. The lack of correlation is consistent with the premise that faking orgasm is inherently insincere communication (Frith, 2018) and that higher sexual satisfaction relies on honest and anxiety-free communication (Herbenick et al., 2018). These findings suggest that faking orgasm is unlikely to enhance relationship well-being (Csányi et al., 2022) or its long-term maintenance.

#### 5.2.2 Reasons for having sex questionnaire

A complex and multi-faceted relational framework emerges regarding sexual motivations, allowing for a deeper exploration of the various reasons behind orgasm faking and their relationship with motivations for engaging in different sexual activities.

Within the YSEX?-HSF Personal Goal Attainment main scale, which represents egocentric sexual motivations, only the Boosting Self-esteem subscale correlates with certain forms of faking. It can be inferred that subscales such as Seeking Novelty, Infidelity, Impulsiveness, Control and Power, or Revenge are not related to women’s orgasm faking. The weak correlation between self-centered sexual motivation and orgasm faking may be attributed to the prevalence of this type of motivation in sexual activities among men rather than women (Meskó et al., 2022). Our results indicate that women tend to fake orgasm during both receiving oral sex and sexual intercourse to elevate their arousal and avoid negative feelings, thereby potentially increasing their self-esteem. Many women experiencing orgasm difficulties encounter distress and anxiety during sex with their partners (Rowland et al., 2019), which not only negatively affects their quality of life but also their self-esteem, therefore, they may resort to faking orgasm as a coping mechanism (Erdõs et al., 2023).

The YSEX?-HSF Relational Reasons main scale, including the Self-affirmation, Care, and Happiness seeking subscales, demonstrates an association with orgasm faking for arousal-enhancing reasons during sexual intercourse. Despite there being no biological difference between clitoral and vaginal orgasm, societal norms often lead women to desire orgasms solely through vaginal penetration (Hoy et al., 2021). According to findings by Impett et al. (2005) and Meston and Buss (2007), participation in sexual activities can increase not only physical pleasure but also foster emotional closeness and commitment at a relational level for women. Although many women report satisfaction without achieving orgasm, there is normative pressure to experience orgasm to feel feminine and normal (Nicolson and Burr, 2003), motivating women to enhance their chances of reaching orgasm by elevating their sexual arousal through self-perception mechanisms (Barnett et al., 2019).

The sexual motivational characteristic of women (Meskó et al., 2022) is evident in the present sample, with the strongest associations related to orgasmic experience found in the Sex as Coping subscale in both oral and sexual intercourse scenarios. Individuals engaging in sexual intercourse to alleviate emotional deficits are more likely to fake orgasm to avoid their own negative feelings or to end the sexual activity. Sexual acts occurring within negative emotional contexts, such as feeling a lack of love or loneliness, play a crucial role in the development of sexual distress (Frith, 2018), enabling individuals to end sexual acts out of habit or obligation without relational tension (Thomas et al., 2017). Our research also confirms that in the sexual domain, women tend to subordinate themselves to their partners and expected norms (Elmerstig et al., 2008; Meskó et al., 2022; Young, 2006), often engaging in sexual intercourse motivated by subordination— prioritizing their partners’ needs. We observed the strongest associations with orgasm faking for sexual adjournment, especially in the case of faking during sexual intercourse.

### 5.2.3 Comparing women who reported faking orgasm to women who had not

The tertiary aim of current study was to assess the replicability of the original version of the Hungarian Faking Orgasm Scale (FOS) with 56 items. Comparing women who reported faking an orgasm with those who reported never faking an orgasm allows us to distinguish between the characteristics of these groups.

As predicted, significant differences were observed in the Distancing and Caring Partner dimensions of the Women’s Sexual Working Models Scale in both oral and intercourse situations. Women who reported faking orgasm exhibited higher scores in the Distancing subscale, aligning with previous research indicating that faking contributes to indifference toward sexual activity and the partner, resulting in decreased relationship satisfaction over time (Csányi et al., 2022). Conversely, women who reported not faking orgasm tend to report greater satisfaction with their sex lives and perceive their partners as more caring, valuing emotional connection over physical satisfaction (Fahs and Plante, 2016). However, contrary to our expectations, no significant differences were observed in behaviors related to negative self-schema and sexual anxiety between the two groups.

Regarding sexual motivations, as expected, significant differences were found between women who reported faking orgasm and those who did not in the YSEX?-HSF Sex as Coping and the Personal Goal Attainment main scales. Women who reported faking orgasm were less likely to engage in sexual activities for relational bonding but rather as a means to enforce their own sexual interests or cope with emotional difficulties. Although men tend to engage in sexual activities for self-centered reasons compared to women (Meskó et al., 2022), our results suggest that, in general, women who reported faking orgasm are more likely to participate in both oral sex and sexual intercourse for personal reasons, such as boosting self-esteem, compared to women who reported not faking orgasm.

Using sex as a coping mechanism is a motivation predominantly observed in women (Meskó et al., 2022), and our findings indicate this is significantly more characteristic of women who reported faking orgasm. This aligns with the mate-retaining elements of coping motivation (Meskó et al., 2022), with faking orgasm itself considered a mate retention strategy (Csányi et al., 2022), allowing women to increase their partner’s commitment to the relationship (Impett et al., 2005; Meston and Buss, 2007).

## 6 Limitations and future directions

The instrument used in current study has several limitations. Firstly, the results and correlations should be interpreted within certain boundaries. While the instrument endeavors to uncover the reasons behind orgasm faking to the best of its ability, it cannot comprehensively address this multifaceted process. For instance, it does not delve into the socio-cultural conditions that may influence women’s sexual behavior, nor does it consider potential health issues that could physically hinder reaching orgasm. Despite these limitations, when used in conjunction with other supplementary questionnaires, the Hungarian brief form of the Faking Orgasm Scale for Women (FOS-24) emerges as a highly reliable measurement tool.

Secondly, although the overall sample used in the study was large and relatively diverse, it did not undergo representativeness testing. For instance, the samples may have omitted asexual respondents who might lack interest in participating in a study on sexual behavior. Additionally, self-reported data, while valuable, are inherently susceptible to biases such as social desirability or recall inaccuracies, which could influence participants’ responses.

Thirdly, the questionnaire was specifically designed to understand the motives behind orgasm faking among heterosexual women only. It is imperative to develop a measure suitable for studying the faking motives of women with sexual orientations other than heterosexual. This could be achieved by supplementing the instructions, thereby ensuring a broader and more comprehensive understanding of female sexuality.

Finally, future research should explore the scale’s applicability across diverse populations and settings. Testing its validity and reliability in different cultural and socio-demographic contexts would provide further insights into its universal utility.

## 7 Conclusion

The current study significantly contributes to the understanding of faking orgasm, revealing the intersection of sexual behavior, motivation, and working models. The findings suggest key pathways for future research and offer valuable insights applicable in clinical practice with women and couples. Professionals, including psychologists, therapists, gynecologists, psychiatrists, can gain a deeper insight into how sexual working models and sexual motives influence decision-making processes within relationships. By understanding the impact of sexual working models and motivations on sexual decision-making, providers can pose clinically relevant questions and explore different implications for patients. Insights from the study may uncover patterns such as feeling obligated to engage in sexual activity despite a lack of desire or resorting to faking orgasm. Without a precise understanding of sexual decision-making processes, providers risk pathologizing common behaviors that may serve important psychological and relational functions.

## Data Availability

The datasets presented in this study can be found in online repositories. The names of the repository/repositories and accession number(s) can be found below: https://osf.io/96emq/?view_only=8eca7faedc0d4f67848c029eac2a0070.
