# Supplementary material for: Psychometric evaluation of the abbreviated Hungarian Faking Orgasm Scale for Women
Source: Front Psychol. 2024 Dec 12;15:1513959. doi: 10.3389/fpsyg.2024.1513959 (PMC11670484; doi:10.3389/fpsyg.2024.1513959)
Supplement: Supplementary file 1 [file Table_1.DOCX]

Psychometric Evaluation of the Abbreviated Hungarian Faking Orgasm Scale for Women

Supplementary Materials

**Table S1** Discrimination (a) parameter values for all items in the item response analysis, separately for scales 2

Hungarian Brief Version of the Faking Orgasm Scale for Women (FOS-24) 4

**Table S2** Factor Loadings of the Hungarian Brief Version of Faking Orgasm Scale for Women (FOS-24) 6

**Table S2A** Loading of the four factors of the Oral Sex subscales of the Faking Orgasm Scale For Women Hungarian Brief Version 6

**Table S2B** Loading of the four factors of the Sexual Intercourse subscales of the Faking Orgasm Scale for Women Hungarian Brief Version 7

**Table S3** The results of the Mann Whitney U test for women who reported having faking orgasm during receiving oral sex and sexual intercourse in terms of sexual working models and sexual motivations (N = 768) 8

**Table S4.A** Sexual working models and motivation of women who reported having ever faked an orgasm compared to women who reported having never faked an orgasm during receiving oral sex; descriptives (M, SD) for WSWMS, YSEX?-HSF and results of analyses of Mann Whitney U-test in 24-item and 56-item Hungarian Faking Orgasm Scale for Women 9

**Table S4B** Sexual working models and motivation of women who reported having ever faked an orgasm compared to women who reported having never faked an orgasm during sexual intercourse; descriptives (M, SD) for WSWMS, YSEX?-HSF and results of analyses of Mann Whitney U-test in 24-item and 56-item Hungarian Faking Orgasm Scale for Women 11

**Table S1** Discrimination (a) parameter values for all items in the item response analysis, separately for scales

| Scale | Item nr | *a* | Scale | Item nr | *a* |
| --- | --- | --- | --- | --- | --- |
| Oral Sex subscales | | | Sexual Intercourse subscales | | |
| Altruistic Deceit | 6 | 2.657 | Altruistic Deceit | 1 | 2.190 |
|  | **7** | 3.420 |  | 2 | 2.268 |
|  | **8** | 4.423 |  | **3** | 3.249 |
|  | **9** | 3.642 |  | **4** | 3.181 |
|  | 10 | 2.742 |  | 5 | 1.777 |
|  | 11 | 2.151 |  | 8 | 2.539 |
|  | 12 | 3.118 |  | **9** | 2.926 |
|  | 13 | 1.904 |  | 10 | 1.453 |
|  | 14 | 1.552 |  | 11 | 2.929 |
| Insecure Avoidance | **1** | 3.811 |  | 12 | 2.557 |
|  | **2** | 2.007 |  | 13 | .623 |
|  | 3 | 1.157 |  | 14 | 2.161 |
|  | **4** | 2.276 |  | 16 | 2.194 |
| Elevated Arousal | **16** | 2.287 |  | 18 | .674 |
|  | **17** | 1.964 | Fear and Insecurity | 6 | 1.119 |
|  | **18** | 3.833 |  | **7** | 2.316 |
|  | 19 | .614 |  | 21 | 1.734 |
|  | 20 | 1.667 |  | **22** | 3.033 |
| Fear of Dysfunction | 5 | .520 |  | 23 | 2.064 |
|  | **15** | 3.201 |  | 24 | 1.996 |
|  | **21** | 2.755 |  | **25** | 3.138 |
|  | **22** | 2.506 |  | 26 | 1.917 |
|  |  |  |  | 27 | 2.120 |
|  |  |  |  | 28 | 1.555 |
|  |  |  | Elevated Arousal | 20 | 1.079 |
|  |  |  |  | 29 | 2.777 |
|  |  |  |  | 30 | 2.972 |
|  |  |  |  | 31 | .466 |
|  |  |  |  | **32** | 3.201 |
|  |  |  |  | **33** | 3.684 |
|  |  |  |  | **34** | 3.295 |
|  |  |  | Sexual Adjournment | **15** | 3.560 |
|  |  |  |  | **17** | 2.155 |
|  |  |  |  | **19** | 1.559 |

*Note*: Retained items are in bold.

Hungarian Brief Version of the Faking Orgasm Scale for Women (FOS-24)

A NŐI SZÍNLELET ORGAZMUS KÉRDŐÍV RÖVID MAGYAR VÁLTOZATA

**Instrukció**: Kérjük, szánjon rá egy percet, hogy átgondolja azt a kapcsolatát, amelyben Ön orgazmust (szexuális kielégülést) színlelt. Most pedig töltse ki az alábbi kérdőívet. Kérjük, válassza ki egy korábbi vagy jelenlegi kapcsolatát, amely egyezik a következő szempontokkal.

Ez a kérdőív az Ön és partnere viselkedésének felmérésére irányul. Tehát nem egy tudáspróbáról van szó, nincsenek jó és rossz válaszok. Kérjük, válaszoljon minden egyes kérdésre olyan pontosan, amilyen pontosan csak tud. Olvassa el valamennyi kérdést gondosan és jelölje be a megfelelő választ.

Egy-egy fogalom meghatározása eltérő lehet a különböző személyek számára. Amellett, hogy tiszteletben szeretnénk tartani az Ön meghatározását, fontosnak tartjuk a kutatási célok elérése érdekében, hogy egységes fogalmakkal rendelkezzünk. Ezért kérjük, – még ha nem is ért egyet velük – hogy használja a következő meghatározásokat, miközben válaszol a kérdésekre.

**orgazmus**: felfokozott izgalmi állapot; a szexuális izgalommal együtt járó feszültség robbanásszerű csúcspontja, amelyet a férfiaknál az ondó kilövellése kísér, a nőknél pedig vaginális izom-összehúzódások; más szavakkal „elélvezni” vagy „elmenni”.

**színlelt orgazmus**: annak megjátszása vagy tettetése, hogy valakinek orgazmusa van (pedig nincs), és a cél érdekében izom-összehúzódások és/vagy hangos visszajelzés alkalmazása.

Köszönjük együttműködését és bizalmát, amely igen megtisztelő számunkra.

ORÁLIS SZEX: Kérjük, hogy a következő kérdésekre olyan értelemben válaszoljon, hogy Ön fogadja az orális szexet (amit úgy határozunk meg, mint a nemi szervek izgatása a partner szája, nyelve által)

** Ha a „SOHA” választ adta a „Milyen gyakran színlel orgazmust orális szex alatt?” kérdésre, akkor kérjük, hogy lépje át a következő szakaszt (kérdéseket).

Amikor orgazmust színlel az orális szex alatt, milyen rendszeresen teszi ezt a következő okokból?

| 1 | 2 | 3 | 4 | 5 |
| --- | --- | --- | --- | --- |
| soha | alkalmanként | néha | gyakran | mindig |

1. Mert kínosan érzi magát az orális szex közben?
2. Mert fizikailag kényelmetlenül érzi magát orális szex közben?
3. Mert zavarban van?
4. Mert szeretné díjazni a partnere erőfeszítését?
5. Szeretné ezzel partnere önértékelését fokozni?
6. Ettől partnere sikeresebbnek fogja érezni önmagát?
7. Mert szégyelli magát, hogy nem képes elérni orgazmust?
8. Hogy „beindítsa” (felizgassa saját magát)?
9. Mert az gondolja, hogy ez szexi?
10. Hogy fokozza saját érdeklődését a szexuális élményben?
11. Mert aggódik, hogy nem képes elérni az orgazmust?
12. Hogy elkerülje a szexuális és nőgyógyászati egészségével kapcsolatos rossz érzéseket amiatt, hogy nincs valódi orgazmusa?

SZEXUÁLIS KÖZÖSÜLÉS: Kérjük, a következő kérdésekre olyan értelemben válaszoljon, hogy valakivel szexuálisan közösül (a közösülést úgy határozzuk meg, mint a pénisz behatolása a vaginába).

**Megjegyzés: Ha a „SOHA” választ adta a „Milyen gyakran színlel orgazmust szexuális közösülés közben?” kérdésre, akkor kérjük, hogy lépje át a következő szakaszt (kérdéseket).

Amikor orgazmust színlel szexuális közösülés közben, milyen rendszeresen teszi ezt a következő okokból?

| 1 | 2 | 3 | 4 | 5 |
| --- | --- | --- | --- | --- |
| soha | alkalmanként | néha | gyakran | mindig |

1. Hogy a partnere sikeresnek érezze önmagát?
2. Hogy megóvja partnerét a csalódottságtól, ha Önnek nincs valódi orgazmusa?
3. Mert aggódik, hogy nem képes elérni az orgazmust?
4. Mert a partnere boldogabb lenne, ha Önnek orgazmusa lett volna közösülés közben?
5. Mert abba akarja hagyni a szexet, de szeretné elkerülni, hogy a partnere kényelmetlenül érezze magát a jövőben?
6. Mert szeretne aludni?
7. Mert egyszerűen nem élvezi az együttlétet?
8. Mert szégyelli magát, hogy nem képes elérni orgazmust?
9. Hogy elkerülje, hogy rosszul érzi magát, ha nincs orgazmusa?
10. Hogy fokozza szexuális élményének izgalmát?
11. Hogy fokozza saját vágyát a közösülés közben?
12. Hogy fokozza a szexuális aktus erősségét?

Kiértékelés:

| Női Színlelt Orgazmus Kérdőív rövid változatának (FOS-24) skálái | | Tételek |
| --- | --- | --- |
| Orális alskála |  |  |
|  | OSAD | 4, 5, 6 |
|  | OSIA | 1, 2, 3 |
|  | OSEA | 8, 9, 10 |
|  | OSFD | 7, 11, 12 |
| Közösüléses alskála |  |  |
|  | SIAD | 1, 2, 4 |
|  | SIFI | 3, 8, 9 |
|  | SIEA | 10, 11, 12 |
|  | SISA | 5, 6, 7 |

*Megjegyzés*: Orális szex alskálák: Altruista színlelés (OSAD); Bizonytalan elkerülés (OSIA); Fokozott izgalom (OSEA); Félelem a diszfunkciótól (OSFD); Szexuális közösülés alskálák: Altruista színlelés (SIAD); Félelem és bizonytalanság (SIFI); Fokozott izgalom (SIEA); Szexuális elhalasztás (SISA)

**Table S2** Factor Loadings of the Hungarian Brief Version of Faking Orgasm Scale for Women (FOS-24)

**Table S2A** Loading of the four factors of the Oral Sex subscales of the Faking Orgasm Scale For Women Hungarian Brief Version

|  | | | | | | *95% Confidence Interval* | |
| --- | --- | --- | --- | --- | --- | --- | --- |
| **Factor** | *Item nr* | *Std. Estimate* | *Std. Error* | *z-value* | *p* | *Lower* | *Upper* |
| **Altruistic Deceit** | 4 | .859 | .042 | 2.275 | < .001 | .776 | .942 |
|  | 5 | 1.074 | .038 | 28.080 | < .001 | .999 | 1.149 |
|  | 6 | 1.047 | .039 | 27.035 | < .001 | .971 | 1.123 |
| **Insecure Avoidance** | 1 | 1.010 | .048 | 21.188 | < .001 | .916 | 1.103 |
|  | 2 | .718 | .053 | 13.440 | < .001 | .613 | .822 |
|  | 3 | 1.081 | .060 | 17.970 | < .001 | .963 | 1.199 |
| **Elevated Arousal** | 8 | .989 | .046 | .046 | < .001 | .898 | 1.080 |
|  | 9 | .832 | .052 | 16.037 | < .001 | .730 | .933 |
|  | 10 | 1.035 | .046 | 22.708 | < .001 | .946 | 1.124 |
| **Fear of Dysfunction** | 7 | 1.324 | .048 | 27.776 | < .001 | 1.230 | 1.417 |
|  | 11 | 1.057 | .053 | 2.080 | < .001 | .954 | 1.160 |
|  | 12 | .756 | .055 | 13.826 | < .001 | .649 | .863 |

**Table S2B** Loading of the four factors of the Sexual Intercourse subscales of the Faking Orgasm Scale for Women Hungarian Brief Version

|  | | | | | | *95% Confidence Interval* | |
| --- | --- | --- | --- | --- | --- | --- | --- |
| **Factor** | *Item nr* | *Std. Estimate* | *Std. Error* | *z-value* | *p* | *Lower* | *Upper* |
| **Altruistic Deceit** | 1 | .962 | .041 | 23.616 | < .001 | .882 | 1.041 |
|  | 2 | 1.051 | .041 | 25.869 | < .001 | .972 | 1.131 |
|  | 4 | .923 | .043 | 21.521 | < .001 | .839 | 1.007 |
| **Fear and Insecurity** | 3 | 1.115 | .039 | 28.388 | < .001 | 1.038 | 1.192 |
|  | 8 | 1.120 | .038 | 29.290 | < .001 | 1.045 | 1.195 |
|  | 9 | 1.020 | .043 | 23.796 | < .001 | .936 | 1.104 |
| **Elevated Arousal** | 10 | 1.058 | .032 | 32.847 | < .001 | .995 | 1.121 |
|  | 11 | 1.088 | .032 | 33.763 | < .001 | 1.025 | 1.151 |
|  | 12 | 1.088 | .031 | 35.160 | < .001 | 1.028 | 1.149 |
| **Sexual Adjournment** | 5 | 1.285 | .060 | 21.393 | < .001 | 1.167 | 1.403 |
|  | 6 | .681 | .043 | 15.705 | < .001 | .596 | .766 |
|  | 7 | .743 | .048 | 15.415 | < .001 | .649 | .838 |

**Table S3** The results of the Mann Whitney U test for women who reported having faking orgasm during receiving oral sex and sexual intercourse in terms of sexual working models and sexual motivations (N = 768)

|  |  | *24-item Hungarian Faking Orgasm Scale for Women (current study)* | | *56-item Hungarian Faking Orgasm Scale for Women (Csányi et al., 2024)* | |
| --- | --- | --- | --- | --- | --- |
|  |  | *Receiving oral sex* | | | |
|  |  | *U* | *ES* | *U* | *ES* |
| **WSWMS** | **Guilt and Shame** | 72918 | .009 | 67224 | .013 |
|  | **Maintain the Bond** | 73039 | .008 | 67461 | .009 |
|  | **Distancing** | 61221*** | .168 | 57906*** | .149 |
|  | **Caring Partner** | 66521* | .096 | 61760* | .093 |
|  | **Excitement** | 71455 | .029 | 67483 | .009 |
| **YSEX-HSF** | **Personal Goal Attainment** | 64175** | .128 | 57464*** | .156 |
|  | **Relational Reasons** | 71498 | .029 | 63777 | .063 |
|  | **Sex As Coping** | 59663*** | .189 | 50613*** | .256 |
|  |  | *Sexual intercourse* | | | |
|  |  | *U* | *ES* | *U* | *ES* |
| **WSWMS** | **Guilt and Shame** | 72918 | .009 | 72918 | .009 |
|  | **Maintain the Bond** | 73039 | .007 | 73039 | .007 |
|  | **Distancing** | 61221*** | .168 | 61221*** | .168 |
|  | **Caring Partner** | 66521* | .096 | 66521* | .096 |
|  | **Excitement** | 71455 | .029 | 71455 | .029 |
| **YSEX-HSF** | **Personal Goal Attainment** | 64175** | .128 | 64175** | .128 |
|  | **Relational Reasons** | 71498 | .029 | 71498 | .028 |
|  | **Sex As Coping** | 59663*** | .189 | 59663*** | .189 |

*Note:* * *p* < .05, ** *p* < .01, *** *p* < .001; WSWMS = Women’s Sexual Working Models Scale; YSEX?-HSF = Reasons for Having Sex Questionnaire Hungarian Short Form.

**Table S4.A** Sexual working models and motivation of women who reported having ever faked an orgasm compared to women who reported having never faked an orgasm during receiving oral sex; descriptives (M, SD) for WSWMS, YSEX?-HSF and results of analyses of Mann Whitney U-test in 24-item and 56-item Hungarian Faking Orgasm Scale for Women

|  |  | *24-item Hungarian Faking Orgasm Scale for Women (current study)* | | | | *56-item Hungarian Faking Orgasm Scale for Women (Csányi et al., 2024)* | | | |
| --- | --- | --- | --- | --- | --- | --- | --- | --- | --- |
|  |  | *Women Who Reported Having Ever Faked an Orgasm During Receiving Oral Sex* | *Women Who Reported Having Never Faked an Orgasm During Receiving Oral Sex* |  |  | *Women Who Reported Having Ever Faked an Orgasm During Receiving Oral Sex* | *Women Who Reported Having Never Faked an Orgasm During Receiving Oral Sex* |  |  |
|  |  | *M (SD)* | *M (SD)* | *U* | *ES* | *M (SD)* | *M (SD)* | *U* | *ES* |
| **WSWMS** | **Guilt and Shame** | 7.40 (3.15) | 7.67 (3.70) | 72918 | .009 | 7.27 (2.77) | 7.70 (3.77) | 67224 | .013 |
|  | **Maintain the Bond** | 25.34 (4.38) | 24.98 (5.01) | 73039 | .008 | 25.41 (4.22) | 25.00 (4.98) | 67461 | .009 |
|  | **Distancing** | 8.29 (3.94) | 7.34 (3.75) | 61221*** | .168 | 8.42 (4.11) | 7.44 (3.68) | 57906*** | .149 |
|  | **Caring Partner** | 17.81 (3.00) | 18.23 (2.89) | 66521* | .096 | 17.71 (3.03) | 18.21 (2.89) | 61760* | .093 |
|  | **Excitement** | 16.45 (3.16) | 16.21 (3.39) | 71455 | .029 | 16.25 (3.38) | 16.37 (3.23) | 67483 | .009 |
| **YSEX-HSF** | **Personal Goal Attainment** | 44.21 (12.7) | 42.10 (14.01) | 64175** | .128 | 45.05 (13.04) | 42.02 (13.54) | 57464*** | .156 |
|  | **Seeking novelty** | 8.09 (3.01) | 7.53 (3.05) | 66043* | .103 | 8.19 (2.96) | 7.58 (3.07) | 59927** | .120 |
|  | **Conformity** | 3.41 (1.2) | 3.51 (1.25) | 69186* | .060 | 3.53 (1.36) | 3.42 (1.15) | 66952* | .017 |
|  | **Infidelity** | 3.64 (1.58) | 3.54 (1.56) | 71519 | .028 | 3.71 (1.71) | 3.52 (1.48) | 64342 | .055 |
|  | **Impulsiveness** | 6.15 (2.95) | 5.75 (2.96) | 67114* | .088 | 6.18 (2.97) | 5.81 (2.95) | 62709 | .079 |
|  | **Revenge** | 3.77 (1.81) | 3.62 (1.84) | 68670* | .067 | 3.94 (2.03) | 3.56 (1.69) | 59931*** | .120 |
|  | **Seeking sensation** | 8.02 (3.25) | 7.61 (3.45) | 67881 | .078 | 8.07 (3.29) | 7.66 (3.39) | 63160 | .072 |
|  | **Control and power** | 4.45 (2.41) | 4.29 (2.63) | 68245* | .073 | 4.55 (.51) | 4.26 (2.53) | 62136* | .087 |
|  | **Boosting self-esteem** | 6.68 (3.34) | 6.24 (3.56) | 65645** | .108 | 6.88 (3.42) | 6.20 (3.46) | 58742** | .137 |
|  | **Relational Reasons** | 88.77 (19.73) | 87.37 (2.56) | 71498 | .029 | 89.88 (18.53) | 87.00 (2.99) | 63777 | .063 |
|  | **Sexual desire** | 12.00 (3.10) | 11.50 (3.51) | 68845 | .065 | 12.00 (3.06) | 11.60 (3.47) | 64929 | .046 |
|  | **Commitment** | 1.99 (3.78) | 1.76 (3.87) | 70924 | .036 | 11.28 (3.66) | 1.64 (3.90) | 61454* | .097 |
|  | **Physical attraction** | 9.37 (3.23) | 9.35 (3.60) | 73546 | 9.51e-4 | 9.22 (3.21) | 9.43 (3.54) | 65481 | .038 |
|  | **Relaxation** | 8.09 (3.54) | 7.90 (3.56) | 71526 | .028 | 7.95 (3.43) | 8.01 (3.62) | 67695 | .006 |
|  | **Intimacy** | 12.12 (2.92) | 12.26 (3.01) | 70565 | .041 | 12.17 (2.81) | 12.20 (3.05) | 66350 | .025 |
|  | **Excitement** | 8.66 (3.22) | 8.55 (3.54) | 71978 | .022 | 8.87 (3.18) | 8.45 (3.50) | 63427 | .068 |
|  | **Self-affirmation** | 6.57 (3.28) | 6.64 (3.03) | 71121 | .034 | 6.78 (3.17) | 6.51 (3.14) | 64459 | .053 |
|  | **Care** | 9.18 (3.45) | 8.84 (3.42) | 69523 | .055 | 9.59 (3.35) | 8.67 (3.44) | 57531*** | .155 |
|  | **Happiness seeking** | 11.80 (3.00 | 11.58 (3.10) | 70405 | .044 | 12.02 (2.89) | 11.49 (3.13) | 61582** | .095 |
|  | **Sex As Coping** | 4.70 (13.48) | 36.74 (12.34) | 59663*** | .189 | 42.12 (13.68) | 36.67 (12.25) | 50613*** | .256 |
|  | **Mitigating emotional deficit** | 7.05 (3.57) | 6.63 (3.83) | 66614* | .095 | 7.38 (3.74) | 6.52 (3.66) | 58276*** | .144 |
|  | **Compulsion and avoidance** | 5.58 (2.55) | 4.80 (2.07) | 60420*** | .179 | 5.73 (2.51) | 4.86 (2.19) | 52878*** | .223 |
|  | **Utilitarianism** | 3.58 (1.59) | 3.41 (1.36) | 69775 | .052 | 3.69 (1.77) | 3.38 1.27) | 62454** | .083 |
|  | **Coping with relational conflicts** | 7.01 (3.29) | 6.22 (3.23) | 62496*** | .151 | 7.26 (3.40) | 6.22 (3.16) | 55563*** | .184 |
|  | **Submissiveness** | 6.70 (3.05) | 6.32 (2.83) | 67823* | .078 | 6.91 (3.06) | 6.27 (2.86) | 58040*** | .147 |
|  | **Coping with partner’s emotional demands** | 4.32 (2.16) | 4.01 (1.69) | 69334 | .058 | 4.47 (2.22) | 3.98 (1.73) | 59172*** | .131 |
|  | **Mate retention** | 6.46 (3.48) | 5.35 (3.04) | 59519*** | .192 | 6.68 (3.52) | 5.43 (3.09) | 53223*** | .218 |

*Notes:* * *p* < .05 ** *p* < .01 *** *p* < .001; WSWMS = Women’s Sexual Working Models Scale; YSEX?-HSF = Reasons for Having Sex Questionnaire, Hungarian Short Form (YSEX?-HSF).

**Table S4B** Sexual working models and motivation of women who reported having ever faked an orgasm compared to women who reported having never faked an orgasm during sexual intercourse; descriptives (M, SD) for WSWMS, YSEX?-HSF and results of analyses of Mann Whitney U-test in 24-item and 56-item Hungarian Faking Orgasm Scale for Women

|  |  | *24-item Hungarian Faking Orgasm Scale for Women (current study)* | | | | *56-item Hungarian Faking Orgasm Scale for Women (Csányi et al., 2024)* | | | |
| --- | --- | --- | --- | --- | --- | --- | --- | --- | --- |
|  |  | *Women Who Reported Having Ever Faked an Orgasm During Sexual Intercourse* | *Women Who Reported Having Never Faked an Orgasm During Sexual Intercourse* |  |  | *Women Who Reported Having Ever Faked an Orgasm During Sexual Intercourse* | *Women Who Reported Having Never Faked an Orgasm During Sexual Intercourse* |  |  |
|  |  | *M (SD)* | *M (SD)* | *U* | *ES* | *M (SD)* | *M (SD)* | *U* | *ES* |
| **WSWMS** | **Guilt and Shame** | 7.40 (3.15) | 7.67 (3.70) | 72918 | .009 | 7.40 (3.15) | 7.67 (3.70) | 72918 | .009 |
|  | **Maintain the Bond** | 25.34 (4.38) | 24.98 (5.01) | 73039 | .007 | 25.34 (4.38) | 24.98 (5.01) | 73039 | .007 |
|  | **Distancing** | 8.29 (3.94) | 7.34 (3.75) | 61221*** | .168 | 8.29 (3.94) | 7.34 (3.75) | 61221*** | .168 |
|  | **Caring Partner** | 17.81 (3.0) | 18.23 (2.89) | 66521* | .096 | 17.81 (3.0) | 18.23 (2.89) | 66521* | .096 |
|  | **Excitement** | 16.45 (3.16) | 16.21 (3.39) | 71455 | .029 | 16.45 (3.16) | 16.21 (3.39) | 71455 | .029 |
| **YSEX-HSF** | **Personal Goal Attainment** | 44.21 (12.70) | 42.10 (14.01) | 64175** | .128 | 44.21 (12.70) | 42.10 (14.01) | 64175** | .128 |
|  | **Seeking novelty** | 8.09 (3.01) | 7.53 (3.05) | 66043* | .103 | 8.09 (3.01) | 7.53 (3.05) | 66043* | .128 |
|  | **Conformity** | 3.41 (1.20) | 3.51 (1.25) | 69186* | .060 | 3.41 (1.20) | 3.51 (1.25) | 69186* | .060 |
|  | **Infidelity** | 3.64 (1.58) | 3.54 (1.56) | 71519 | .028 | 3.64 (1.58) | 3.54 (1.56) | 71519 | .028 |
|  | **Impulsiveness** | 6.15 (2.95) | 5.75 (2.96) | 67114* | .088 | 6.15 (2.95) | 5.75 (2.96) | 67114* | .088 |
|  | **Revenge** | 3.77 (1.81) | 3.62 (1.84) | 68670* | .067 | 3.77 (1.81) | 3.62 (1.84) | 68670* | .067 |
|  | **Seeking sensation** | 8.02 (3.25) | 7.61 (3.45) | 67881 | .078 | 8.02 (3.25) | 7.61 (3.45) | 67881 | .077 |
|  | **Control and power** | 4.45 (2.41) | 4.29 (2.63) | 68245* | .073 | 4.45 (2.41) | 4.29 (2.63) | 68245* | .073 |
|  | **Boosting self-esteem** | 6.68 (3.34) | 6.24 (3.56) | 65645* | .108 | 6.68 (3.34) | 6.24 (3.56) | 65645* | .108 |
|  | **Relational Reasons** | 88.77 (19.73) | 87.37 (2.56) | 71498 | .028 | 88.77 (19.73) | 87.37 (2.56) | 71498 | .028 |
|  | **Sexual desire** | 12.00 (3.10) | 11.50 (3.51) | 68845 | .064 | 12.00 (3.10) | 11.50 (3.51) | 68845 | .064 |
|  | **Commitment** | 1.99 (3.78) | 1.76 (3.87) | 70924 | .036 | 1.99 (3.78) | 1.76 (3.87) | 70924 | .036 |
|  | **Physical attraction** | 9.37 (3.23) | 9.35 (3.60) | 73546 | 9.51e-4 | 9.37 (3.23) | 9.35 (3.60) | 73546 | 9.51e-4 |
|  | **Relaxation** | 8.09 (3.54) | 7.90 (3.56) | 71526 | .028 | 8.09 (3.54) | 7.90 (3.56) | 71526 | .028 |
|  | **Intimacy** | 12.12 (2.92) | 12.26 (3.01) | 70565 | .041 | 12.12 (2.92) | 12.26 (3.01) | 70565 | .041 |
|  | **Excitement** | 8.66 (3.22) | 8.55 (3.54) | 71978 | .022 | 8.66 (3.22) | 8.55 (3.54) | 71978 | .022 |
|  | **Self-affirmation** | 6.57 (3.28) | 6.64 (3.03) | 71121 | .034 | 6.57 (3.28) | 6.64 (3.03) | 71121 | .034 |
|  | **Care** | 9.18 (3.45) | 8.84 (3.42) | 69523 | .055 | 9.18 (3.45) | 8.84 (3.42) | 69523 | .055 |
|  | **Happiness seeking** | 11.80 (3.0) | 11.58 (3.1) | 70405 | .043 | 11.80 (3.0) | 11.58 (3.1) | 70405 | .043 |
|  | **Sex As Coping** | 4.70 (13.48) | 36.74 (12.34) | 59663*** | .189 | 4.70 (13.48) | 36.74 (12.34) | 59663*** | .189 |
|  | **Mitigating emotional deficit** | 7.05 (3.57) | 6.63 (3.83) | 66614* | .095 | 7.05 (3.57) | 6.63 (3.83) | 66614* | .095 |
|  | **Compulsion and avoidance** | 5.58 (2.55) | 4.80 (2.07) | 60420*** | .179 | 5.58 (2.55) | 4.80 (2.07) | 60420*** | .179 |
|  | **Utilitarianism** | 3.58 (1.59) | 3.41 (1.36) | 69775 | .052 | 3.58 (1.59) | 3.41 (1.36) | 69775 | .052 |
|  | **Coping with relational conflicts** | 7.01 (3.29) | 6.22 (3.23) | 62496*** | .151 | 7.01 (3.29) | 6.22 (3.23) | 62496*** | .151 |
|  | **Submissiveness** | 6.70 (3.05) | 6.32 (2.83) | 67823* | .078 | 6.70 (3.05) | 6.32 (2.83) | 67823* | .078 |
|  | **Coping with partner’s emotional demands** | 4.32 (2.16) | 4.01 (1.69) | 69334 | .058 | 4.32 (2.16) | 4.01 (1.69) | 69334 | .058 |
|  | **Mate retention** | 6.46 (3.48) | 5.35 (3.04) | 59519*** | .191 | 6.46 (3.48) | 5.35 (3.04) | 59519*** | .191 |

*Notes:* * *p* < .05 ** *p* < .01 *** *p* < .001; WSWMS = Women’s Sexual Working Models Scale; YSEX?-HSF = Reasons for Having Sex Questionnaire, Hungarian Short Form (YSEX?-HSF)
